# Antecedents for online food delivery platform leading to continuance usage intention via e-word-of-mouth review adoption

**DOI:** 10.1371/journal.pone.0290247

**Published:** 2023-08-17

**Authors:** Saleh Yahya Alghamdi, Sumeet Kaur, Karishma M. Qureshi, Ali Saeed Almuflih, Naif Almakayeel, Saleh Alsulamy, Mohamed Rafik N. Qureshi

**Affiliations:** 1 Department of Industrial Engineering, College of Engineering, King Khalid University, Saudi Arabia, Abha, Saudi Arabia; 2 Area the Quantitative Techniques and Operations Management, FORE School of Management, New Delhi, India; 3 Department of Mechanical Engineering, Parul Institute of Technology, Parul University, Waghodia, India; 4 Architecture and Planning Engineering Department, College of Engineering, King Khalid University, Abha, Saudi Arabia; Universidad Autonoma de Chihuahua, MEXICO

## Abstract

The focus of hospitality initially was on ambience and novelty to attract customers. With the rise of the digital revolution, the hospitality industry has also undergone significant change. Long-distance travel at the workplace, odd working hours, and a variety of food options have driven people staying in Indian metropolises towards online food delivery (OFD) services. The popularity of OFD services has risen because of their practicality, simplicity, and a rise in consumer confidence in digital payments. Specifically, for the food industry, digitalization has opened new horizons to capture customers. The competition is not among the big brands, but big brands are competing with homemakers who run tiffin services, and street food hawkers who claim to provide traditional Dhaba-style food and fast food. The customers are loaded with unlimited options to choose the food in terms of price, cuisine, quality, etc. The present research examines the associations between service quality of OFD services, perceived ease of use, and word-of-mouth review adoption, leading to expectation confirmation modeling. The path analysis was carried out using data from 500 Indian respondents residing in Tier-I cities who have been using OFD services regularly. The research outcome shows that servqual has a positive influence on perceived ease of use and confirmation. Additionally, it encourages continued usage intentions because of its favorable impact on the adoption of e-word-of-mouth reviews.

## I. Introduction

**Fig 1:**
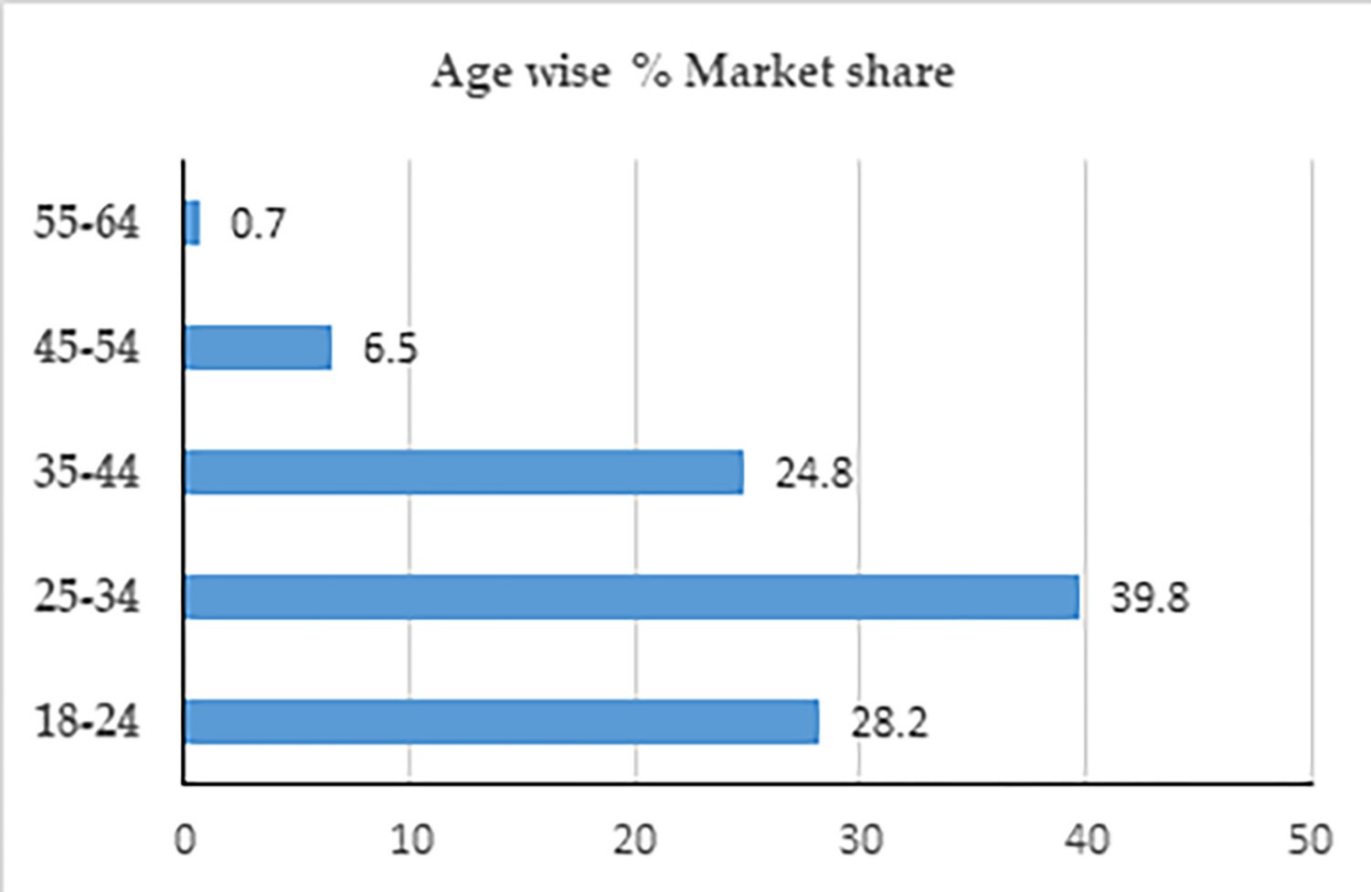
Age-wise % market share of OFD meal service.

Online food ordering involves ordering food from a local restaurant or food delivery provider through a web page or app. Many working professionals around the world, especially those who reside in cities, now rely heavily on the online food delivery (OFD) system. The OFD is an innovative method of business delivery and is gaining popularity, particularly among students who move out of the house to study or young, active, and employed people. Online food ordering is a promising business growing among young Indians. One of the Indian census data indicates that more than 50% of its population is below the age of 25 and more than 65% below the age of 35. Based on the survey data, 65% of online food delivery orders belong to Indians under 35 years of age (Statista, 2019). Thus, the Indian online food business shows tremendous customer base potential among young Indians, as can be seen in Fig 1.

**Fig 2:**
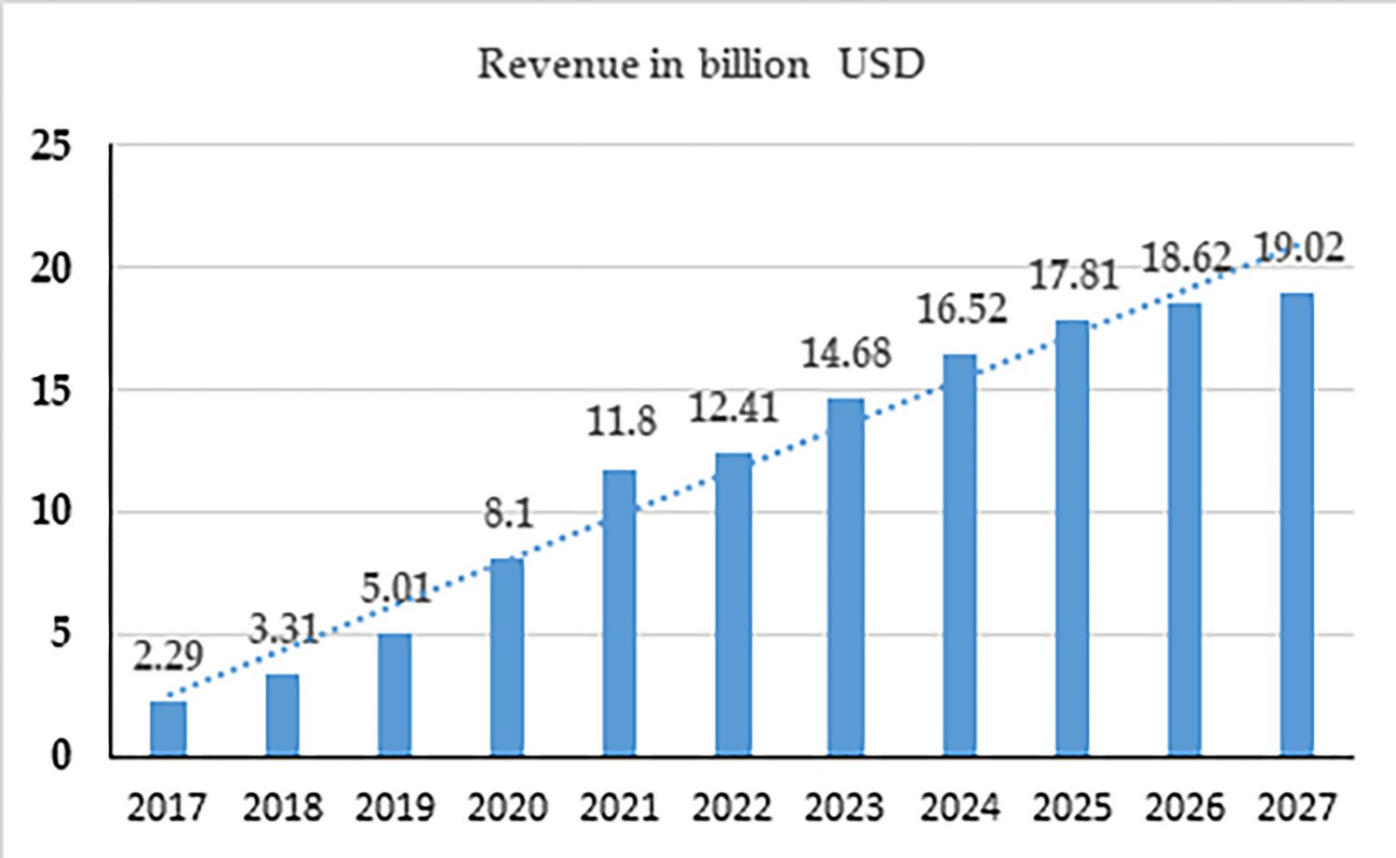
Revenue in billions of USD.

Most businesses nowadays have ventured into online services to increase their market share and revenue. Restaurants are one such business that has seen tremendous growth on online platforms as the demand for OFD has been increasing steadily. Between 2022 and 2028, the Indian OFD market is anticipated to increase at a 33 percent compound annual growth rate (CAGR) (Renub,2019). Revenue in the OFD market is projected to reach US $14.68 bn in 2023, and further, it is projected to reach a volume of US $ 19.02 bn by 2027, as shown in Fig 2.

Because of a rise in the number of new online business enterprises, the OFD industry has become more competitive. The OFD business faces challenges as consumers’ expectations are increasing and they seek more delivery and time convenience with increasing anxiety. OFD’s service platform offers a dynamic and agile supply chain that fulfills the customers’ needs by supplying ordered food in the best possible manner in terms of speed, taste, proper packaging, and value for their money. Thus, the OFD service platform offers better choices and customer experience to its clients. However, because of the thin margin in the food delivery business [1], the OFD service platform makes a persistent attempt by connecting customers to restaurant owners through their customers’ feedback and customer experience to improve its food quality and business strategies along with service. The frequent changes in business service delivery boost the customer experience and customer satisfaction, which leads to loyalty [2]. Customer loyalty has a favorable impact on a business’s ability to grow and earn profits [3]. As a result, providing high-quality services is crucial for keeping clients happy. Keeping service quality in focus, various empirical and structural models based on service quality have been prepared and tested to know customer satisfaction and repurchase intentions [4].

In food e-commerce, trust plays an important role. Due to its prominent position and substantial volume of peer-generated contents, it is also seen as an important element in social commerce (s-commerce) [5]. In a business, customer value influences the business. Customer value shows how much value a customer thinks a product or service has. Maximizing this value can give a company a competitive edge if it builds it into its business model. Each product or service consists of the perceived value leading to the purchase of a product or service, giving rise to the brand image to exhibit positive relationships [6]. The necessity of product or service differentiation is a fundamental requirement for the survival and expansion of businesses, which is why it is included in business strategies [7]. The definition of service quality is arbitrary and relies on customer perception and expectation. Thus, the prevailing gap between expectations and performance is the key factor in determining customer satisfaction [8–10].

Apart from food quality, several service system attributes like reliability, responsiveness, assurance, and empathy play a vital role in determining whether a consumer will choose a given OFD service platform or not. Additional sub-factors like information quality, service experience, relative advantage, response time, accessibility, and interface are important in providing customer satisfaction. Customer satisfaction, food quality, and OFD service platform quality positively influence purchase intention [11].

With the cutthroat competition and increasing food service demand in society, the continuous assessment of online self-service is mandatory to be in the market. Several studies have taken place engaging in the investigation of online services using salient scales, face-to-face service evaluation, and online service assessment with real-time feedback. OFD service companies like Zomato, Swiggy, Uber Eat, Magicpin, etc. provide internet-enabled web or mobile apps that connect small and big hoteliers and customers. Aside from that, some restaurant owners deliver independently by fulfilling the food supply chain in the best way possible through their employees. In either case, the success of a business depends on the service quality of OFD.

Various factors are responsible for influencing customer behavior while placing an order for OFD services. According to a study, consumer perceptions and online food ordering are positively correlated [12]. One of the Indian OFD studies [13] concluded that overall attitudinal and behavioral variations influence OFD usage. There is a significant direct influence of servqual attributes on perceived ease-of-use (PEOU) and confirmation, whereas indirect influence is believed to be on continuance usage (CU) of OFD service. The mediating role of e-word-of-mouth review adoption (EWOMRA) is also playing a significant role in influencing CU; hence, further investigation of these attributes is necessary to reveal their relationship. Further, looking at the steady increase in OFD demand influencing food e-commerce businesses, additional investigation into OFD is necessary to address the following research questions: What are the antecedents of the service quality of OFD? How does servqual drive PEOU? How does servqual drive the confirmations? What is the role of PEOU and confirmation towards CU? How does confirmation influence word-of-mouth review adoption? How does the eWOM review adoption lead to CU? Thus, the present study investigates the role of service quality of the OFD service platform when delivered, leading to influences towards PEOU, confirmation, and ultimately CU following a word-of-mouth review adoption. Therefore, the present study investigates these attributes using the variance-based partial least square technique. The study confirms the direct effect of servqual on PEOU and confirmation towards CU. It also confirms that EWOMRA also plays a mediating role in CU. The present research will contribute to gaining customer loyalty through an effective strategy.

The research in question is evidenced by the following: Section 2 illustrates the theoretical framework of the present research by reviewing a wide spectrum of literature to formulate research hypotheses. The research approach used to create the current empirical analysis and structural relationship model is illustrated in Section 3. In Section 4, results from the suggested model are described. The important finding is covered in the final section.

## II. Literature review

Customers are attracted to online food delivery systems due to their popularity and convenience. The term "behavioral intention" refers to this behavior. There are few studies found on Indian OFD examining their behaviour while opting for the OFD platforms [12], consumer attitudes, and behaviour intentions [13]. Behavioral intention may be related to someone’s likelihood of behaving or their propensity to use the service again [14]. The prior study contrasted the immediate effects of OFD e-service and food quality resulting in loyalty. They also demonstrated the indirect influence of perceived value and customer satisfaction on loyalty [15]. Social media sites like Facebook and Instagram are used for e- commerce, which also influences decision-making in online purchases [16,17]. Customer loyalty to business-to-consumer (B2C) in e-commerce is significantly influenced by online customer satisfaction, EWOM and online trust [18]. Online shoppers’ satisfaction is a key factor in determining their trust, repurchase, and WOM intentions [19]. Further, it has been established that customer trust is positively associated with customer purchase intention [20].

How OFD consumers make their buying decisions is very significant; hence, consumer perception was studied. It was further investigated how consumers are affected by OFD businesses like Swiggy, Zomato, Uber Eat, Magicpin, etc., [21]. Using mixed research techniques, the study carried out data analysis and created the scale to judge the OFD service quality scale [22]. They further revealed OFD industry-related twenty key variables and embedded into their scale. The study further revealed six important dimensions of created scale: assurance, traceability, security, operation-maintenance, food quality, and cleanliness.

To provide high-quality service, the offered service must adhere to various significant variables, including customer satisfaction, timeliness in food delivery, service applicability, capability for service replication, and service standards [23]. Various attributes like customer trust, customer satisfaction, and loyalty in e-commerce services are predicted to be important determinants of the success of online businesses [5]. The social When factors influencing consumers’ decisions to buy food online were examined, it was found that one of the most crucial factors was service quality [18]. Additionally, several studies found that providing excellent OFD services will boost OFD operators’ customer happiness and loyalty [24,25]. OFD services use urban transit to reduce the hardship of customers navigating through crowded cities [26]. According to a study, there is a favorable correlation between system functioning, assurance, dependability, and customer happiness [22].

The OFD sector’s main service to customers is food delivery, which is done by its deliverymen or third-party service providers. Hence, OFD initiatives must follow through with quality checks at every stage of their processes to meet quality standards and customer expectations. From the beginning of the food preparation process until it is in the customer’s hands, the delivery of the OFD service plays a critical role. Thus, food delivery promptness, maintenance of food quality [27], and conversations between delivery men and customers also form important service quality criteria that decide the future ordering of customers. Thus, it demonstrates that an integral part of the OFD process is service quality [28]. A past study of the food and beverage industry investigated the effect of customer service and its consequences on loyalty, and satisfaction [29]. Several significant factors like food quality, food hygiene, and systematic service to deliver convenience are integral parts of OFD service quality. These qualities should not be disregarded in the OFD assessment and must be appropriately controlled. The OFD operators must develop the standard to meet the service quality needs.

In the following subsection, we provide the various attributes that contribute to service quality on the OFD platform. We also provide characteristics of each attribute to develop the influence on the PEOU, confirmation, and CU. E-word-of-mouth review adoption also plays a contributing role toward CU.

## III. Servqual–peu-confirmation- word-of-mouth review adoption—cu model

Service quality is how well customer needs are met through a service [8]. Customer perceptions of the quality of the services provided in the context of OFD may have an impact on customer satisfaction and loyalty [15]. Parasuraman et al. [8] propose five quality factors (reliability, responsiveness, tangibles, empathy, and assurance) that connect particular service attributes to customer expectations of quality. They also incorporated these factors into a well-accepted service quality measuring instrument known as servqual. Later, Parasuraman et al. [8] came out with a service quality measuring scale for customers transacting online. The empirical study of Indian OFD found ‘convenience’ and ‘ease of information’ influence ‘consumer satisfaction’ and boost ‘consumer intentions. The study also found that convenience also significantly affects ‘consumer intentions’ [12]. The study examining the online service quality of retailers by 539 users revealed a positive association between service quality and satisfaction, leading to loyalty [30]. The service quality positively correlates with its service usefulness, service user-friendliness, and trust, leading to continuous usage [31,32]. The past study also examined the importance of online consumer reviews and revealed various factors that encourage customers to take up the service [33]. The study evaluating customer buying intention found that customer satisfaction has a positive association between food quality and OFD service [24]. The previous study investigated the role of trust on a social networking site. The study revealed that trust has a positive influence on purchase intentions. It also encourages information seeking, which in turn improves purchase intentions [5]. Another study investigated the role of customer loyalty in the e-commerce business. Online customer satisfaction is influenced by EWOM and online trust [18]. One of the studies revealed that customer trust, satisfaction, and loyalty have a significant role to play in e-commerce services; hence, they must be considered while assessing online business success [34]. A recent study involving online consumers investigated consumer satisfaction and revealed that satisfaction influences trust, repurchase, and EWOM intentions. Thus, customer trust is influenced by consumer satisfaction and enables repurchase intentions [19]. In s-commerce, the user experience plays a significant role in the perceived usefulness and perceived ease of use. It further influences trust [16].

According to the comprehensive review of the literature, there are several scales available for assessing the quality of e-services. The various scales available in the literature are given in Table 1.

**Table 1:** Service quality scales for E-service.

Name of the developed scale	Researchers	Dimensions included in the scale
SERVQUAL	[8]	Reliability, empathy, tangibles, assurance, assurance, and responsiveness
SERVPERF	[35]	Service quality, Food quality, price/value (SQ) responsiveness
SITEQUAL	[36]	Aesthetic design, Ease of use, interactive, responsiveness and processing speed,
E-S-QUAL	[37]	Efficiency, fulfillment, availability of system, and privacy
E-S-QUAL (revised)	[38]	Delivery speed, efficiency, fulfillment, reliability, availability of system, and privacy
e-SELFQUAL	[24]	Food quality, e-SELFQUAL (control, convenience, customer service fulfillment) service fulfillment

**Fig 3:**
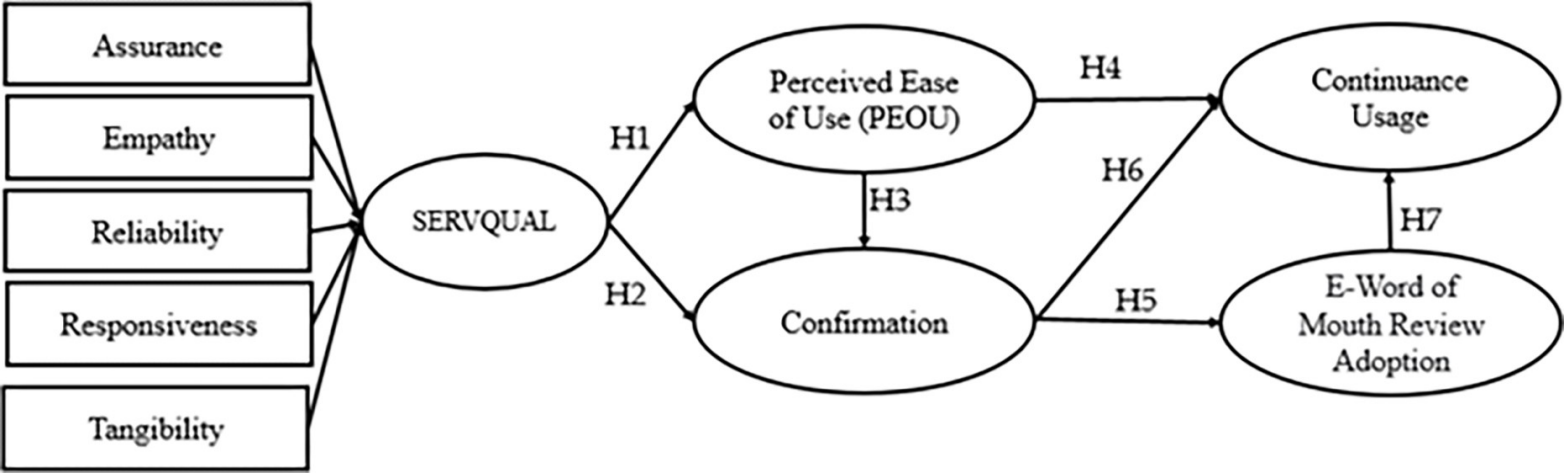
Servqual–PIU-Confirmation- Word of mouth- CU model. Based on the discussed conceptual model, the following hypotheses are constructed: H1: Service quality attainment is positively linked with PEOU. H2: Service quality attainment is positively linked to confirmation. H3: PEOU has a positive association with confirmation. H4: PEOU is positively linked with CU. H5: Confirmation has a positive association with e-WOM review adoption. H6: Confirmation is positively linked with CU. H7: EWOMRA is positively linked with CU.

The present study proposes the integration of servqual, perceived intention of usage, confirmation, and word-of-mouth review adoption towards the continuance intention of service users, as shown in Fig 3.

## IV. Attributes contributing to ofd service quality

The quality of the OFD service provider is determined by how the customer’s OFD service is provided. The way service provider gives the necessary details regarding the menu, its ingredients, a price list, discounts offered, and assurances about the food’s OFD service. The quality of an OFD service, speed of accessing webpages, information transmission, and response time to a query are important for customer satisfaction.

Researchers and professionals test to see if the OFD service meets the needs of the users. A careful analysis of the literature has revealed the need for additional studies to determine the consequences of OFD services and user behavior. Further, in the OFD service evaluation, there are varying perspectives and staggered opinions about the OFD service assessment criteria. Many researchers have attempted to evaluate the OFD service and how it relates to consumer behavior. Such earlier attempts are shown in Table 2.

**Table 2:** Research studies on the OFD in the recent past.

Researchers	Measurement scale	Research domain
[39]	Perceived ease of use, orientation for timesaving, convenience motivation, security, and privacy	Malaysian studies
[24]	Quality of foody, e-SELFQUAL consisting of control, customer service fulfillment, and convenience	American OFD service
[25]	Price, service quality, the attitude of the delivery person, Menu and variety of restaurants, delivery time, condition of food delivered,	OFD service of Bangladesh
[40]	User habit, users’ Performance expectations, Impulse buying tendency, Congruity with self-image, mindfulness	American OFD service
[22]	Food quality maintainability, Food hygiene, service reliability, assurance, security, transparency, system operation	OFD customers in Taipei City

## V. Research methodology

### A. Measurement scales

To evaluate the suggested research model, an online poll was conducted. Care was taken to include those OFD users who had regularly used online food ordering for the previous six months and had accessed it at least twice per month. A five-point Likert scale was used to evaluate the items for all the variables, with 1 standing for "strongly disagree" and 5 for "strongly agree." The scales were modified from established scales, and the item’s language was changed to fit the context of online meal ordering. By removing items from a reflecting multi-item scale, the scale purification procedure was undertaken [41,42]. The scale purifications helped fulfill the compliance. The 40-item survey was reduced to 28 due to the findings of the exploratory factor analysis (EFA).

### B. Sampling

For the current study, the purposive sampling technique was employed to collect the data. It is a non-random sampling technique, as it gives flexibility to the researcher to find people who are willing to provide the information based on knowledge or experience. In the population of the study, only those respondents who had previously ordered meals online were chosen using the filter-question. The respondents who used online food delivery services from any of the OFD service providers (e.g., Swiggy, Zomato, Uber Eat, Maigcpin, etc.) for at least 6 months and accessed it at least twice in a month were requested to respond to the questionnaire. The questionnaire was distributed through various electronic channels and personal contacts. Similarly, feedback was also gathered.

### C. Data collection

The data were gathered from November 1 through December 31, 2022. Only 780 of the total 1200 participants filled out the survey. The minimum sample size needed for the study is 5 respondents per item, resulting in a sample size of 140, and the preferred sample size is 20 respondents per item, resulting in a sample size of 560. The study was completed with 500 valid samples, which was sufficient for the analysis, yielding a response rate of 41.6 percent. The participants provided the necessary participation consent. They voluntarily participated without any financial or non-financial aid. The participants were allowed to exit the study without any restrictions. Care was taken in deciding the response, excluding criteria for further analysis. The responses were excluded from further analysis based on the following two response exclusion criteria: The first criterion was to exclude responses with similar markings for all items. This indicates the seriousness of the respondents’ responses to the various questions. The second exclusion criterion selected was an incomplete response.

### D. Data analysis and model testing

The present model has both reflective and formative constructs. To create and validate the model, a partial least squares approach to structural equation modelling (PLS-SEM) was used. Further, it is better to apply PLS-SEM as the data violates the assumption of multivariate normality. The survey data may be modelled using this robust approach while analysing the feedback data [43]. It has been recommended to use at least 10 times the total variables used in the constructs [44]. The present research considers more data than required. The present research uses data from 500 OFD users. A two-step approach was carried out using the Smart PLS 4. Reliability and validity analyses were used to examine the measurement model. The structural model then proceeded to estimate the connections between its constructs. The importance of path linkages and the model’s goodness of fit were assessed.

## VI. Results

### A. Sample characteristics

The responses were submitted by 278 men and 222 women who used OFD services, according to an analysis of the data. As a result, the proportion of responses was 55.6 percent for men and 44.4 percent for women, respectively. Most respondents in the response samples were younger than 35 years old (79.8%). Table 3 lists the demographic data of the respondents.

**Table 3:** Demographics information.

Demographics	Frequency	Percentage (%)
Gender		
Women	222	44.4
Men	278	55.6
Age Range		
<25	16	3.2
25–30	255	51.0
30–35	128	25.6
35–40	39	7.8
>40	62	12.4
Profession		
Student	67	13.4
Employee	369	73.8
Owning business	47	9.4
Housewife	17	3.4

Employees are most likely to use the OFD service platform, according to demographic statistics i.e. (73.8%), followed by students, owners of businesses, and housewives. The survey item responses demonstrated a higher mean value that was higher than 3.01 and a larger standard deviation value greater than 0.8. The data points reflect a wide range of values when the standard deviation is high [45,46]. Table 4 displays the variable’s mean and standard deviation as well as the sources of the data used in the analysis.

**Table 4:** Descriptive statistics analysis.

Variables	The questionnaire used in the survey	Mean	Std. deviation
Please recall your OFD experience while responding to the following			
Reliability	I believe that OFD service is reliable	3.798	0.8728
	OFD provides service at the promised time	3.867	0.8004
	I trust OFD to deliver on time	3.930	0.8596
Responsiveness	I believe OFD is responsive to my needs	3.495	0.9653
	I believe OFD will provide me with prompt service in the event of any issues.	3.615	0.8875
	The customer service team at OFD will address any concerns that I have	3.652	0.912
Empathy	The OFD team will spot me as a repeat customer (after the first time)	3.428	1.0625
	I think OFD can address the specific needs of each customer	3.348	1.0212
	I liked the available payment alternatives. (e.g., different credit /debit cards/ UPI) at OFD	3.287	1.0312
Tangibility	Employees have a neat, professional appearance	3.12	1.2032
	Up-to-date facilities in terms of website/mobile application	3.01	1.1018
	Visually appealing packaging associated with food delivery service	3.08	1.0032
Assurance	I felt confident about OFD	3.511	1.0409
	OFD makes me feel safe in my transactions	3.652	0.7982
	OFD had answers to all my questions	3.537	1.0429
Perceived ease of use	I had a clear and understandable encounter with an OFD.	3.990	0.8000
	Interacting with an OFD does not require a lot of mental effort	3.903	0.8418
	Overall, I find an OFD to be easy to use.	3.891	0.8416
Confirmation	My experience with using OFD was better than I expected.	3.790	0.9384
	The service level offered by OFD exceeded my expectations.	3.660	0.9513
	Overall, most of my expectations I had for using OFD came true.	3.548	0.9428
E-WOM review adoption	I always read reviews that are presented on the websites before OFD	3.776	0.9081
	The reviews presented on the websites make me confident in OFD.	3.079	1.1067
	I am confused if I don’t read reviews before using the service	3.569	1.003
Continued usage intention	I don’t want to stop using OFD; I intend to keep doing it.	3.861	0.8608
	Instead of employing any other method, I want to keep using OFD.	3.773	0.8802
	I would like to continue using OFD as much as I can if I could.	3.868	0.9432
	Overall, I would continue using OFD	3.945	0.7689

### B. Correlation matrix

A correlation matrix analysis was employed for common method bias. The presence of common method bias can be confirmed if a large value of R is found; thus, any values greater than 0.9 (r > 0.9) confirm the presence of common method bias. During the analysis, it was found that the latent variable correlation was smaller than 0.9. Thus, it provides sufficient evidence for its absence (refer to Table 5).

**Table 5:** Latent variable correlation.

Constructs	AS	EMP	REL	RES	servqual
AS	1				
EMP	0.466	1			
REL	0.535	0.33	1		
RES	0.772	0.467	0.462	1	
servqual	0.859	0.693	0.78	0.832	1

### C. Exploratory factor analysis

The multicollinearity of the variables was examined using principal component analysis (PCA). Principal component analysis was the extraction technique utilized in factor analysis. Eigenvalues larger than 1 were considered when extracting the components. Nine variables explained 78.13 percent of the variation. The data were tested using the Kaiser-Meyer-Olkin (KMO) test to see how well they would support factor analysis. When KMO values are between 0.8 and 1, sampling is suitable. It may be concluded that the sample was adequate because the KMO value for the analysis was 0.862. Small significant level readings for Bartlett’s test of sphericity (less than 0.05) indicate that factor analysis is appropriate for the data.

### D. Confirmatory factor analysis

During the confirmatory factor analysis (CFA), specific observable variables whose weights fell below the standardized regression cutoff of 0.7 were excluded. To reach a high level of fitness, the squared multiple correlations must also be smaller than 0.4 [36]. Various constructs like reliability, assurance, responsiveness, and empathy were found to be linked to service quality, revealing strong multicollinearity. As a result, each element was included in and handled as part of the service quality construct. Reliability and validity were assessed using an analysis based on the criteria of construct reliability of over 0.7 and average variance extracted (AVE) of over 0.5. Table 6 presents the outcomes. The findings revealed that each variable’s AVE was higher than 0.5. Construct reliability was determined to be more than 0.7 for all variables. Additionally, the squared correlation of each component was higher than the number of links among observed independent variables as determined by the AVE of discriminant validity. Consequently, it can be argued that the variables’ ability to discriminate was not hampered [47]. Higher-order constructs are usually based on the relationships, such as reflective or formative, between the model’s constructs [48] and on the number of levels in the model [49]. This can be further classified as a type I model, i.e., reflective-reflective, or a type II model, which is reflective-formative. As the dimensions of servqual are distinct, they do not share a common basis. Hence, it has been assumed that servqual is a reflective-formative type II second-order construct.

**Table 6:** Testing of constructs reliability.

Construct Items	Cronbach’s alpha	Composite reliability (rho_a)	Average variance extracted (AVE)
AS	0.732	0.756	0.657
CNF	0.841	0.841	0.759
CU	0.844	0.854	0.685
EMP	0.844	0.859	0.685
EWOMRA	0.857	0.86	0.777
PEOU	0.783	0.784	0.699
REL	0.857	0.849	0.717
RES	0.797	0.806	0.711
servqual	0.776	0.795	0.538

The repeating indication approach involves describing every component of the underlying lower-order construct to produce a higher-order construct [49]. The five service quality dimensions that make up the current concept of service quality are second-order constructs. The first-order structures for the five dimensions each have a unique set of variables. As a result, all twenty manifest variables of the underlying first-order construct dimensions are used to specify the second-order latent construct servqual. The items have since been utilized to represent primary loadings in first-order latent constructs. The second-order construct taking secondary loadings also makes use of them. The weights for the second-order construct are represented by the path coefficients between the two constructs, first and second. As opposed to estimating the higher- and lower-order constructs individually, repeated indicators estimate all the latent variables concurrently.

### E. Assessment of the measurement model

**Fig 4:**
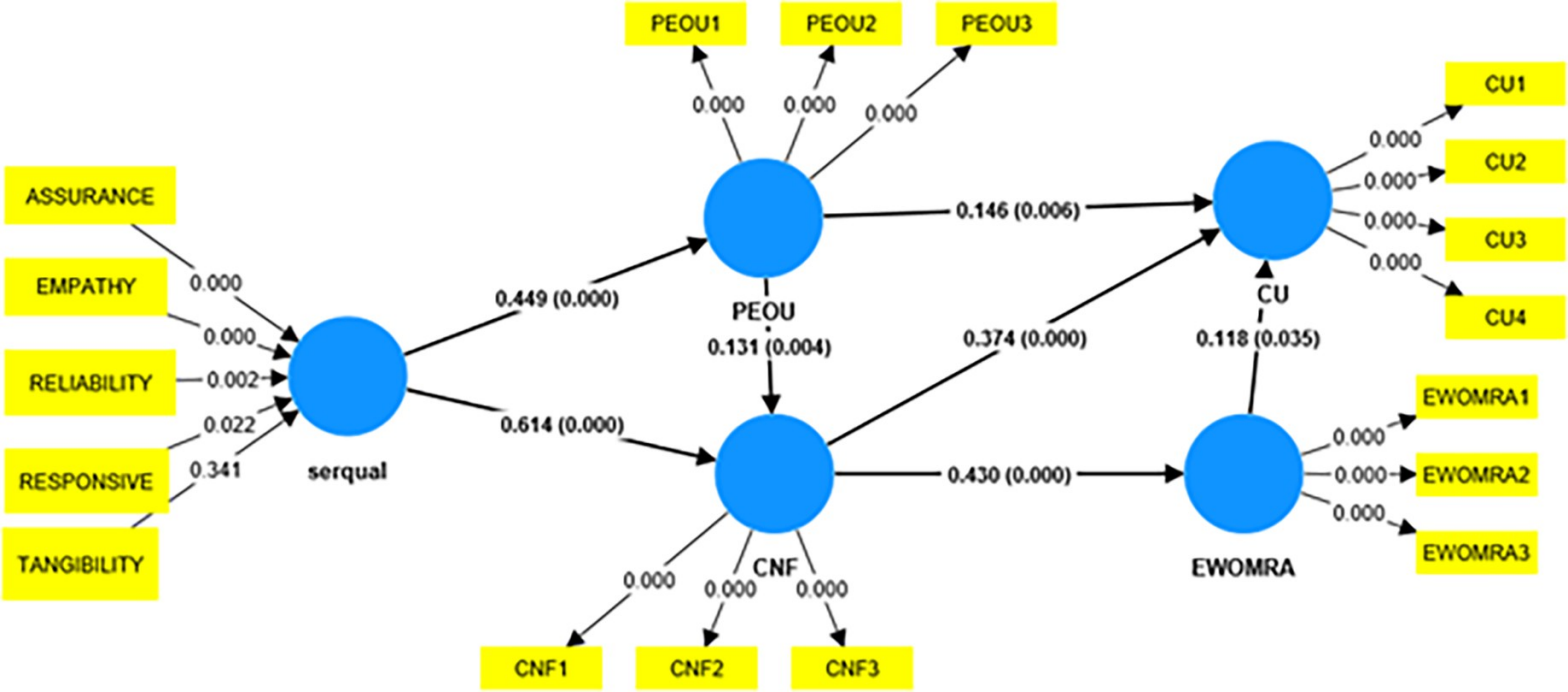
Initial structural equation model.

**Fig 5:**
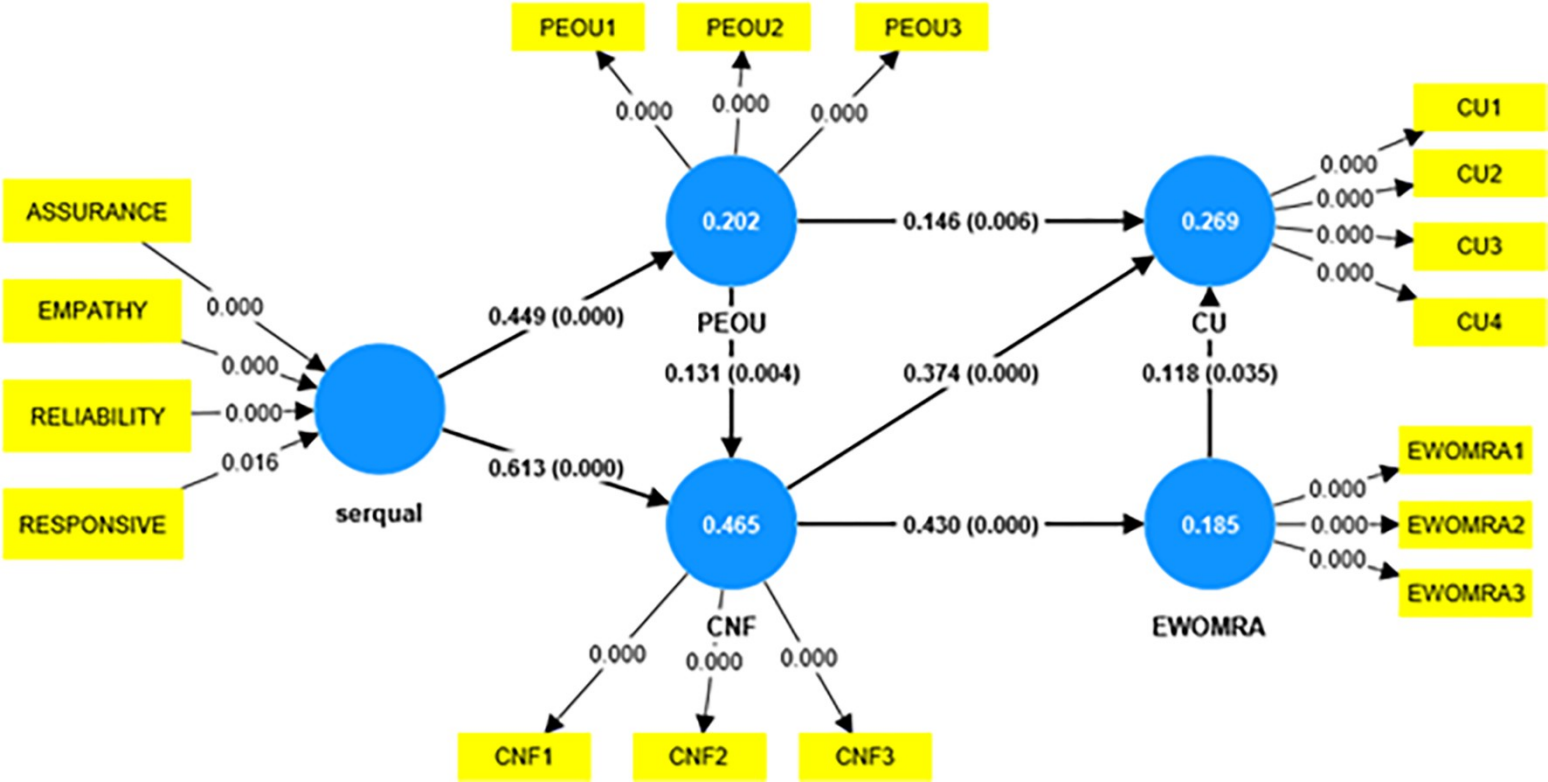
Final structural equation model.

To determine whether the measurement model exhibited convergent validity, factor loadings, composite reliability (CR), and average extracted variance (AVE) were evaluated [47]. The constructs’ internal consistency was evaluated using composite reliability (CR), as described by Hoffmann and Birnbrich [50]. Every latent variable used in this investigation met or exceeded the cutoff value; it also met threshold criterion 0.7 for the CR [43]. Additionally, the factor loadings and the AVE were evaluated to determine the convergent validity of the constructs. Factor loadings between 0.6 and 0.7 are suitable, according to Hair et al., while an AVE value above 0.5 indicates sufficient convergence [47]. Factor loadings and AVEs were above the recommended levels for all latent variables used in this investigation. Table 6 shows various values of CR. Additionally, the loadings and path coefficients of the factors as determined by the PLS algorithm are displayed in Figs 4 and 5.

### F. Reliability and validity (measurement model)

The extracted average variance (AVE), composite reliability, and Cronbach’s alpha (reliability) are shown in Table 6.

Cronbach alphas for each variable ranged from 0.732 to 0.876. The CR value of the latent construct ranged from 0.756 to 0.859, meeting the suggested threshold of 0.7 [51]. The results can be used to verify the questionnaire’s validity and internal consistency. Construct validity was examined to evaluate the measurement model. The average extracted variance (AVE) ranged from 0.538 to 0.777, above the threshold of 0.5 in all cases [52].

To examine discriminant validity, HTMT, as suggested by Hair et al. was used [47]. The HTMT matrix is shown in Table 7, and since every construct was judged to be trustworthy, the validity of the constructs was established. The necessary conditions are satisfied for the measurement model’s approval [53,54]. PLS-SEM uses the standardized root mean square residual (SRMR) as a model fit metric. Hu and Bentler stated that a good match is one where the SRMR is less than 0.08; the SRMR in the current model is 0.067 [55].

**Table 7:** HTMT matrix.

Construct	AS	CNF	CU	EMP	EWOMRA	PEOU	REL	RES
AS								
CNF	0.842							
CU	0.603	0.574						
EMP	0.603	0.574	0.784					
EWOMRA	0.667	0.503	0.377	0.377				
PEOU	0.499	0.5	0.415	0.415	0.421			
REL	0.667	0.503	0.377	0.377	0.671	0.421		
RES	0.791	0.591	0.558	0.558	0.551	0.3	0.551	
servqual	0.757	0.75	0.812	0.841	0.821	0.495	0.812	0.822

For any construct that consists of uncorrelated variables, AVE, CR, and outer loadings have no significance [56]. The importance and relevance of indicator weights and indicator collinearity should be indicated for formative constructs, according to Hair et al. [57]. As stated by Hair et al., the servqual orientation as the reflective-formative construct has been studied [47]. High correlations between the indicators are not feasible for formative measurement methods. A strong correlation between formative items suggests collinearity, which is seen negatively [58]. As a result, the notions of tangibility, assurance, empathy, responsiveness, and reliability were assessed for their potential to predict service quality. Hair et al. state that the VIF cutoff value is less than 5. Table 8 demonstrates that the VIF value for each predictor component was found to be less than 5. Therefore, there is no problem with collinearity between the formative indicators of the constructs [58].

**Table 8:** VIF values.

Construct	VIF
ASSURANCE	1.534
EMPATHY	2.068
RELIABILITY	1.622
RESPONSIVENESS	2.632
TANGIBILITY	2.001

The bootstrapping procedure was performed to examine the significance and applicability of the indicator weights using 5000 samples. The significance of the weight of all indicators was tested. The findings showed that, with the exception of tangibility, all weights were above the suggested value of 0.1 [59]. Table 9 provides the values obtained during the weight test of all constructions.

**Table 9:** Indicator weight.

Construct	Weights of the original sample (O)	P values
AS -> servqual	0.285	0
EMP -> servqual	0.293	0
REL -> servqual	0.396	0.002
RES -> serqual	0.293	0.022
TAN -> serqual	0.092	0.341

The initial measurement model had five latent constructs (reliability, responsiveness, assurance, tangibility, and empathy). Tangibility was insignificant for the model proposed; hence, it was removed from the current analysis. Hence, the service quality was analyzed based on the four significant dimensions, and the model was evaluated. The final hypothesis is reported in Table 10. All the hypotheses were supported.

**Table 10:** Hypothesis results.

Hypothesis	Relationship	Bootstrap sample	Path coefficients	Supported/Not supported
H1	Servqual→PEOU	5000	0.449	Supported
H2	Servqual→Confirmation	5000	0.613	Supported
H3	PEOU→Confirmation	5000	0.131	Supported
H4	PEOU→CU	5000	0.146	Supported
H5	Confirmation→EWOMRA	5000	0.430	Supported
H6	Confirmation→CU	5000	0.374	Supported
H7	EWOMRA→CU	5000	0.118	Supported

## VII. Discussion

The digital revolution has aided many business owners in growing their enterprises. Because of the competition in the restaurant business, good-quality food with lower profit margins compelled the restaurant owners to adopt various strategies to expand their business. OFD’s service quality not only attracts customers but also motivates them to order the food of their choice. The increase in users of OFD services also motivated new players to jump into the restaurant business, especially dealing with takeaway, which poses several challenges to them. The customized food service may help them increase their customer base and profit margin. For OFD service providers, the growing number of restaurant owners presents another issue. Customers frequently change restaurants or OFD service providers because of minor food quality issues. The managers of such OFD service providers need to understand customer satisfaction, their behavior, and their intentions. They must also know how the service quality dimensions influence the customer to gain total satisfaction from the OFD services. The service dimensions like assurance, empathy, responsiveness, and reliability play a significant role in providing satisfaction to OFD users. Therefore, while delivering food, OFD service providers must be mindful of service quality. Customer satisfaction with OFD services is influenced by a number of variables, including food quality, control, customer service, and service fulfilment [24].

The current investigation supports the strong correlation between the service quality dimension and the user’s intention to continue. The results are in line with a study revealing service quality leading to continuous usage [60]. The servqual impact is positively correlated with perceived ease of use and confirmation. Improving the quality of the OFD service will increase the number of OFD users by increasing user satisfaction. The findings are consistent with earlier research [61]. The findings also give present OFD operators systematic information and motivation towards e-commerce survival and growth strategies so that they can design a suitable business policy. The outcome also helps future researchers pinpoint service quality deficiencies more precisely. Therefore, OFD operators offering online services must comprehend the nature, requirements, and relevant aspects of the OFD industries that consumers in this emerging market value highly.

The OFD service providers provide ease of use that leads to continuing to use the OFD services. Customers of OFD services are pleased not only with the food quality but also with the ease of use, which encourages them to continue using the service. It has also been revealed that confirmation leads to word-of-mouth review adoption, which has a positive association with continued usage. Confirmation is prompted due to higher service quality and its perceived ease of use to induce OFD service users to use the OFD services. These two outcomes also encourage OFD service users to finally make their choice based on e-word-of-mouth review adoption. The study also revealed that the intention to use long-term is influenced by considering word-of-mouth reviews. Restaurant managers must maintain the best possible food quality, and timeliness, and coordinate meticulously with OFD service providers for better customer service. The OFD service providers must also maintain a website connecting to the OFD users to offer more value-added content and attempt to deliver on their promises to obtain the expected service quality.

### Theoretical and managerial implications

The present research reveals the direct influence of servqual attributes on PEOU and confirmation towards CU, whereas the mediating role of e-WOM review adoption towards CU. Service quality attributes like assurance, empathy, reliability, responsiveness, and tangibility have been tested for OFD service quality. There is a significant contribution from such dimensions, except for tangibility, in building service quality for customer satisfaction. The OFD service providers, including hotel owners and their practising managers, are required to practise service quality to satisfy the OFD customer and ensure continuous improvement in the OFD business. The present research revealed the direct influence of service quality attributes on the PEOU and confirmation that it leads to CU. The food e-commerce business is experiencing a paradigm shift in online business because of its ease of use and customer satisfaction. The OFD service providers must strive to take up the challenge of providing the highest service quality for their customers’ continued usage.

## VIII. Limitations and directions for future research

The study reveals the impact of the service quality of OFD services on users hailing from an Indian metropolis towards continuance usage intention considering e-word-of-mouth review adoption. A similar study carried out across the globe may end in different results; hence, the results obtained may be generalized with some exceptions. The OFD service opted for may also vary considering the living standards and computer literacy of an individual. The result of such studies may also be influenced by the state of the country, whether it is underdeveloped, developing, or developed. Hence, a comparative study may be carried out considering the differences in cultural, societal, and business conventions. The data for the studies came from OFD users, and the responses of the respondents mostly reflect the quality of the OFD services provided by Zomato, Swiggy, Uber Eat, Magicpin, etc. Future studies may examine additional variables that help explain further variations in customer behavior and satisfaction with online meal ordering. The future studies may also investigate the influence of increasing delivery charges from OFD on CU.

## IX. Conclusion

In the present study, the servqual instrument devised by Parasuraman et al. [8] is employed to assess the relative importance of the quality aspect in relation to how the OFD service platform is perceived. The study revealed the positive relationships between customers’ perceived OFD service quality and its influence on continuance usage. It is also shown that perceived ease of use and confirmation have an impact on service utilization. The study also revealed the positive influence of E-word-of-mouth review adoption to influence continuance usage. So, the OFD service provider, if desires to be in business needs to focus on the parameters of servqual. The role of the OFD service provider is much more than timely delivery. They need to take the onus of the servqual to provide a better experience to customers which ultimately leads to customer retention. The manager may derive a strategy to rank and control the four dimensions of OFD service quality so that customers get satisfaction through service quality. Customers view the value of the quality attribute differently. Consequently, the management of restaurants may try to evaluate such a ranking of service quality dimensions. The ranking will help the managers devise a suitable strategy to evaluate and rank the dimensions. The management of OFD service providers may devise an effective working strategy to cope with the increasing demands and fulfill customers’ anxiety to keep long-term loyalty and customer base.

## Supporting information

S1 Data (XLSX)
